# Veteran peer suicide prevention: A community‐based peer prevention model

**DOI:** 10.1111/sltb.12712

**Published:** 2021-04-20

**Authors:** Sarah Beehler, Carl LoFaro, Carlee Kreisel, Brooke Dorsey Holliman, Nathaniel V. Mohatt

**Affiliations:** ^1^ U.S. Department of Veterans Affairs Rocky Mountain Mental Illness, Research, Education and Clinical Center (MIRECC) Rocky Mountain Regional VA Medical Center Aurora CO USA; ^2^ Department of Family Medicine and Biobehavioral Health University of Minnesota Medical School, Duluth Campus Duluth MN USA; ^3^ Department of Physical Medicine and Rehabilitation University of Colorado Anschutz Medical Campus Aurora CO USA; ^4^ Department of Family Medicine University of Colorado School of Medicine Aurora CO USA; ^5^ Department of Psychiatry Yale School of Medicine New Haven CT USA

## Abstract

**Objective:**

The purpose of this study was to develop a conceptual model of community‐based veteran peer suicide prevention.

**Method:**

We conducted a qualitative study in which semi‐structured interviews were followed by three focus groups. Participants (*n* = 17) were chosen from community‐based organizations who had peers working on veteran suicide prevention; the sample included veteran peers, non‐peers, program managers, and community stakeholders. Interview data were analyzed thematically and inductively to identify key components and subcomponents of veteran peer suicide prevention. A draft model was shared with each focus group to elicit feedback and refine key concepts.

**Results:**

A conceptual model containing nine components and twenty‐six subcomponents was developed. Participants emphasized key organizational, relational, and practical elements needed to achieve positive outcomes. In addition, they described critical contextual and cultural factors that impacted veteran peers’ ability to prevent suicide and promote overall wellness.

**Conclusions:**

Community‐based veteran peer efforts are a promising public health approach to preventing veteran suicide. Provided veteran peers are supported and fully allowed to contribute, these efforts can complement existing clinic‐based efforts. Future research on community‐based veteran peer suicide prevention should document a range of outcomes (e.g., clinical, wellness, financial) and allow for considerable flexibility in peer approaches.

## INTRODUCTION

Veteran suicide is a serious public health concern. Data from 2005 to 17 show that 78,875 veterans died by suicide and that the veteran suicide rate is 1.5 times that of non‐veteran adults, adjusting for age and sex (U.S. Department of Veterans Affairs, 2019). While the VA has launched substantial efforts to prevent veteran suicide, less than half of all veterans are enrolled in VA. Further, only 45% of individuals in the general population who die by suicide have contact with a primary care provider in the month before their death (Luoma et al., 2002). This suggests that clinical approaches to suicide prevention reach a minority of individuals at risk for suicide. Public health and community‐based approaches to preventing veteran suicide are needed in addition to clinic‐based interventions. Peer support strategies are one promising approach to suicide prevention (Huisman & van Bergen, 2019; Pfeiffer et al., 2019). Pfeiffer et al. (2019) described a randomized control pilot study wherein veterans admitted to psychiatric inpatient units for suicidal ideations in the treatment arm of the study met with peer specialists in addition to the usual care. The median number of meetings peer sessions was four over 3 months. The study, measuring the fidelity, feasibility, and acceptability of the intervention, found positive feedback from veteran participants with regard to the peers’ abilities to connect and provide support. However, roles for veteran peers outlined in existing literature are predominantly focused on facilitating formal treatment (Chinman et al., 2010; Greden et al., 2010; Hebert et al., 2008).

Peer support has been defined as “social emotional support, frequently coupled with instrumental support, that is mutually offered or provided by persons having a mental health condition to others sharing a similar mental health condition to bring about a desired social or personal change” (Solomon, 2004, p. 393). For veteran peers, the key similarity is military service and peers may or may not have mental health conditions or other lived experiences (e.g., recovery from substance misuse, housing instability) in common. Peer support exists along a continuum between unidirectional (e.g., with psychiatrists) and reciprocal relationships (e.g., with friends) that can occur intentionally or naturally in either clinical or community settings (Davidson et al., 2006). Depending upon the setting and scope of peer work, peer support services may or may not differ substantially from traditional clinical services (Davidson et al., 2006). In contrast to mutual help and consumer‐run programs (e.g., Alcoholics Anonymous), peer support is asymmetrical, with someone farther along in recovery offering support and/or services to another who is not as far along (Davidson et al., 2006). A review of literature by Bellamy et al. (2017) on peer services found that they produce similar clinical outcomes (e.g., decreased hospitalization rates, decreased symptom severity) to non‐peers and greater impact on recovery‐oriented outcomes (e.g., hope, quality of life, empowerment). It is these recovery outcomes that peers appear particularly well‐suited to addressing. In contrast to non‐peers, peers who can add unique value effectively (e.g., role modeling and disclose appropriately on the basis of shared experience) are especially effective at supporting different aspects of recovery (Bellamy et al., 2017; Davidson et al., 2012). Further, a suicide prevention program delivered by peer support specialists—who receive state training and certification—was determined to be feasible and patients found it acceptable (Pfeiffer et al., 2019). There are, however, certain requirements for successful peer work in this area including personal distance from peers’ suicidality and clear roles and boundaries within the care team (Huisman & van Bergen, 2019). Buddy‐to‐Buddy, a peer‐to‐peer program designed to improve clinical treatment outcomes for returning National Guard members, has hypothesized but not yet tested that peer support would build on cultural connection and reduce risk for suicide by increasing treatment entry and adherence (Greden et al., 2010). This is an example of clinically oriented peer support (i.e., peers work to enhance clinical treatment or formal services) that differs in scope and goals from the community‐based veteran peer efforts we studied. Both types, however, are aligned with the VA approach to veteran suicide prevention.

The VA adopted a public health approach to preventing veteran suicide, The National Strategy for Preventing Veteran Suicide 2018–2028 (U.S. Department of Veterans Affairs, 2018), that includes four key components outlined by the CDC (Centers for Disease Control and Prevention, n.d.): a population‐level focus, primary prevention, scientific rigor, and multidisciplinary collaboration. Public health approaches to reducing suicide complement clinical treatment approaches by adding a broad prevention focus, identifying patterns of suicide, addressing multiple risk factors, and intervening at levels beyond the individual to alter conditions give rise to suicide ideation and attempts (David‐Ferdon et al., 2016; Institute of Medicine, 2002; Satcher, 1998). Like other prevention efforts, this public health approach encompasses three types of suicide prevention that vary in scope and should be matched with individual or group level of risk: universal, selective, and indicated. Universal strategies target an entire population (e.g., all veterans in the United States), and selective strategies target subgroups at risk for suicide (e.g., veterans with substance use disorders) and indicated strategies target high‐risk individuals (e.g., veterans who have attempted suicide; Institute of Medicine, 2002; U.S. Department of Veterans Affairs, 2018).

The multilevel, multicomponent, and multidisciplinary nature of this public health approach complements clinical approaches to reducing suicide, which reach limited segments of veterans in the United States. More community‐based efforts to prevent veteran suicide are needed and, though promising, “peer support is an underused intervention in suicide prevention” (U.S. Department of Veterans Affairs, 2018, p. 29). As outlined in the VA National Strategy, veteran peer support is critical to helping veterans at risk for suicide. Veteran peers can contribute to suicide prevention in a number of ways, including bolstering protective factors (e.g., enhancing sense of connection and belonging, imparting hope and motivation for achieving recovery, fostering a sense of meaning and purpose); promoting physical, mental, emotional, and spiritual wellness as well as support for handling specific stressors; providing culturally sensitive support and challenging stigma; and collaborating with a variety of healthcare providers to link veterans with needed services (e.g., employment, housing) and support aftercare. Thus far, the VA has incorporated veteran peers into some clinic‐ and hospital‐based suicide prevention services, but to date no conceptual model exists to guide community‐based veteran peer suicide prevention work. The purpose of this study was to develop a conceptual model of community‐based peer veteran suicide prevention. We describe a model of veteran peer work that is aligned with public health principles for suicide prevention and can be used in combination with other prevention strategies (Table 1).

**TABLE 1:** Veteran peer model components and subcomponents

Component	Subcomponents
Supportive structure	Formal organization Focus on peer success
Clearly defined role	Boundaries and limitations Scope and responsibilities
Highly effective peers	Skills and knowledge Characteristics and qualities Boundaries Self‐care
Engage veterans in their communities	Show up Respect individual Take long view
Build trusting relationships	Put in the work/time/effort Find shared experiences Work together with integrity
Promote connection	Create opportunities to connect Meaningful connection
Link with resources	
Veteran wellness	Recovery Restored community connection Renewed meaning and purpose Improved mental health/wellness Achieve personal goals Suicide‐specific outcomes
Peer and community impacts	Develop highly effective peers Enrich experience for peers Family impacts Organization and community impacts

## METHOD

This study emerged from a larger community‐based project to prevent suicide among rural veterans, Together With Veterans (TWV; Monteith et al., 2020). Community veteran stakeholders expressed interest in delivering peer‐based suicide prevention services and asked the research team for help. Since there was no model specific to community‐based veteran peer suicide prevention, we decided to learn from the work of existing veteran peer prevention programs to build a conceptual model. The model described here resulted from qualitative interviews conducted with individuals managing different programs. We then conducted a series of focus groups—one each with interview participants, external stakeholders, direct peer service providers—to generate further discussion and receive feedback about our early findings. All data collection took place during 2019.

### Sample

We drew upon contacts from the TWV project to conduct snowball sampling with organizations that met the following inclusion criteria: working with veterans, as well as in mental health and peer support; working in either suicide prevention and/or rural communities; and have been in their current role for at least one year. We contacted nine organizations and conducted semi‐structured phone interviews with program managers from six of them (one organization declined to participate, one did not meet the criteria, and one was unable to schedule an interview). We interviewed one or two staff members (*n* = 7) at each agency.

### Data collection

Each participant completed a semi‐structured interview. Interviews were designed to elicit participant perspectives on the critical aspects of community‐based peer work aimed at preventing veteran suicide. The interview guide included questions on participant background (training, experience), peer services provided, what constitutes “success” or effectiveness in peer work, and elements of the work that may be uniquely or especially important to peer suicide prevention (e.g., ethical conduct, preventing burnout). The interviews also included questions on resources and structural (organizational and community) supports conducive to veteran peer suicide prevention and participants were encouraged to describe the contextual influences (social, community, cultural) on peer work. Two researchers conducted interviews using teleconference software, and recordings were transcribed verbatim by medical research transcriptionists.

### Data analysis

The qualitative analysis was a continuous process beginning with initial interviews and continuing throughout and beyond the data generation period. We used a team‐based, rigorous inductive thematic analysis approach where analysis of the transcripts began with repeated readings to achieve immersion, followed by coding using an emergent approach. Under the guidance of Dr. Dorsey Holliman (qualitative research expert), two members of the study team independently reviewed transcripts and notes to inductively identify ten initial themes. The research team met to review their respective themes and reconcile any discrepancies in theme definitions and applications. This iterative process continued until a final set of nine broad themes representing discrete components of peer work were established, at which point the research team reviewed each transcript to identify all examples of each component. This produced nine extensive lists containing different dimensions of each component. Two coders then independently grouped the examples within each component into like categories and named these subcomponents (i.e., sub‐themes). This was an iterative process and the team met regularly to resolve coding discrepancies through consensus. Subcomponents were coded and categorized until we identified 26 comprehensive and mutually exclusive categories.

Credibility is one of four ways qualitative researchers can establish trustworthiness in data analysis (Lincoln & Guba, 1985). To ensure credibility of the findings, the research team employed multiple strategies. First, we shared our findings with participants to make sure they accurately reflected our conversations and captured important elements of their work. We convened a focus group attended by five of the seven original participants to discuss our draft model and elicit feedback and suggestions, both about the model and implementation recommendations. A second focus group of additional stakeholders provided their reactions and guidance. We then revised the model and presented it via video focus group to a group of five peers from the original organizations to learn how well the model captured their experience, take suggestions, and ideas for next steps. Second, the research team was multidisciplinary with a flattened power structure to ensure all members had equal opportunity to share perspectives and concerns throughout the data collection and analysis process. Third, we kept a detailed audit trail throughout the data collection and analysis process. Finally, we triangulated our findings with existing literature.

## RESULTS

We interviewed seven participants for this study. Four were male and three were female. All were veterans of the United States Air Force, Army, or Navy, with service eras from the Vietnam War to the Post‐9/11 conflicts. All had experience in both management of veteran peer programs and serving as peers. A total of 15 participants attended one of three follow‐up focus groups, including five of the original interviewees. All focus group participants had at least one year of experience with veteran peer work; 13 were veteran peers and program managers, and two were non‐veteran, non‐peer stakeholders.

**FIGURE 1:**
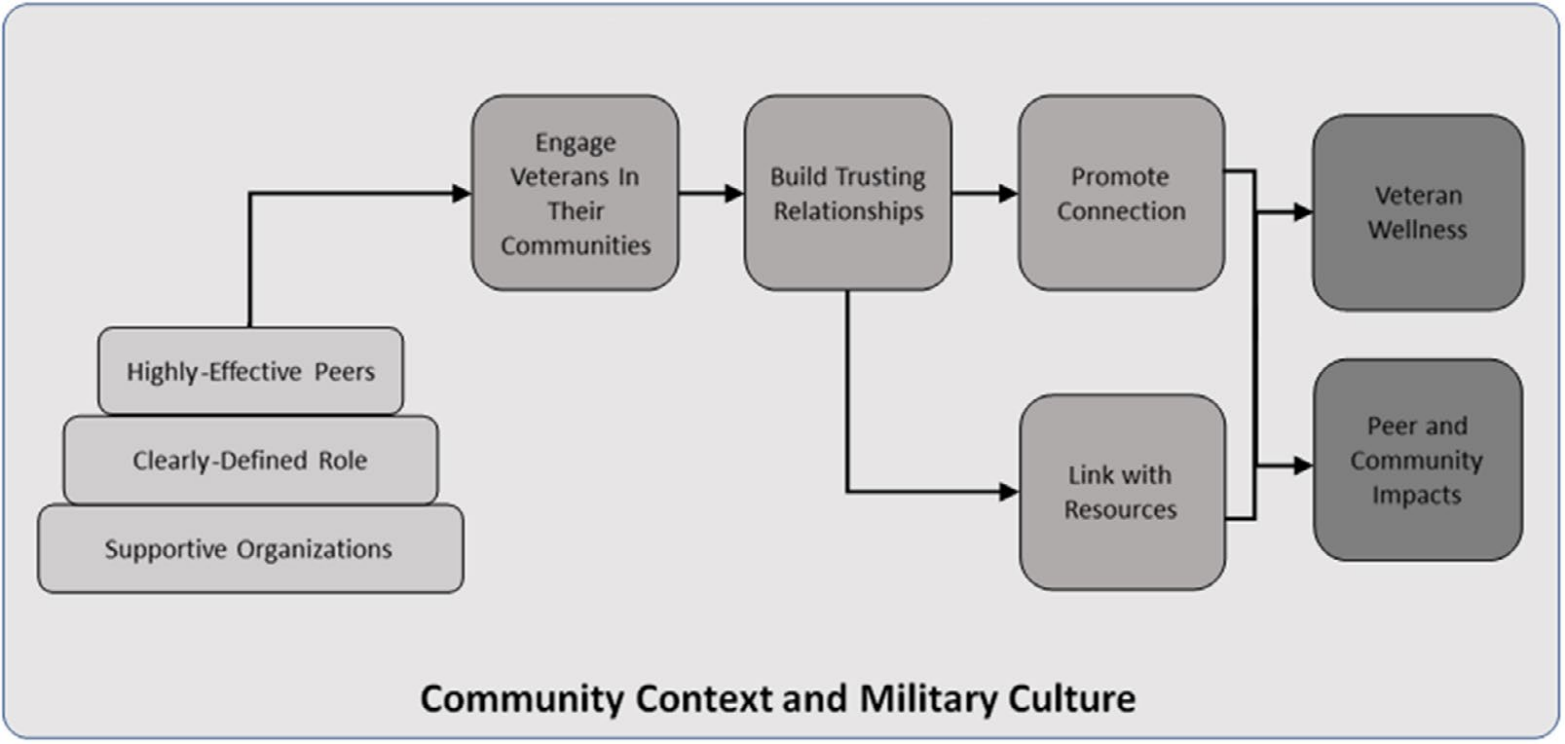
Model of community‐based veteran peer suicide prevention [Colour figure can be viewed at wileyonlinelibrary.com]

Nine components and 26 subcomponents were identified (see Figure 1). Overall, the model reflects a focus on promoting whole health, not just on suicide prevention. Participants repeatedly emphasized the importance of taking a holistic, strengths‐based approach to their peer work and suicide prevention. For example, one participant stated “our concept of peer support is total wellness and suicide is part of that.” From this perspective, suicide prevention is just one important outcome of veteran peer work. Military culture is infused throughout the model, and all of this occurs in the context of broader community and environmental factors. In addition, participants stressed the importance of flexibility and local responsiveness in peer work.

Moving from left to right, the model depicts that highly effective peers operate in clearly defined roles within supportive organizations. They work within communities to proactively engage veterans and build trusting relationships. These relationships then allow for peers to foster connections among vets and/or provide links to tangible resources. All of this produces a wide range of outcomes, for veterans, families, and communities, as well as peers themselves. Below, we provide overviews of the components and subcomponents (see Table 1), and then describe important aspects of community context and military culture. A full description of all subcomponents is beyond the scope of this paper; further information is available from the authors.

### Highly effective peers

Participants described what it meant and took for peers to be effective. Four subcomponents were identified: skills and knowledge, characteristics and qualities, boundaries, and self‐care. Distinctions appeared between personal characteristics and qualities (e.g., proactive, empathic, inclusive) to consider when selecting individuals for peer work and skills and knowledge that can be cultivated (e.g., asking about suicidal thoughts, discussing means reduction) via training and professional development. High‐quality, ongoing training was described as critical to the success of peers. In addition, participants repeatedly emphasized that effective peers establish and maintain appropriate boundaries and communicate about them as needed to veterans. One participant reported that, in his role as a peer, he simply explained to veterans, “I’m not a bank, I’m not a hotel, and I’m not a taxi.” Having clear boundaries was facilitated by having a clearly defined role, and described as critical in managing veteran expectations and protecting peers from burnout. Finally, participants described the importance of peers taking care of themselves (e.g., know limits, be self‐aware, engage support systems) in order to successfully help others.

### Clearly defined role

Participants highlighted the importance of role definition and clarity in peer work. They reported that better defined roles reduced the chances of peers finding themselves in difficult (potentially unethical) situations. Clear roles allow peers to rely on their training and known boundaries. Participants also emphasized that roles for peers need to be defined in relation to the structural or organizational requirements to keep the program going. For example, if reimbursement is essential to sustaining peer programming, completing necessary paperwork in an appropriately confidential manner may be a requirement of the peer role. Two subcomponents were identified: boundaries and limitations and scope and responsibilities. As mentioned previously, understanding the limitations and boundaries of the role was described as critical for peers to work ethically and effectively and to avoid burnout. Examples here included peers understanding they are not crisis counselors and should not work harder than the veterans. Further, participants described peer roles as ranging in scope from narrow (e.g., working only with veterans) to broad (e.g., supporting families, navigating systems, raising community awareness). A number of potential responsibilities were mentioned (e.g., risk assessment, making referrals, navigating systems, community outreach/education, leading groups), but it appeared the most important thing was that organizations be explicit and consistent about both the scope and responsibilities of peer roles.

### Supportive organizations

Participants emphasized the importance of peers being housed within an organizational structure rather than operating independently or informally. There were two subcomponents: formal organization and focus on peer success. Working within a formal organization conferred a number of advantages to peers, including providing legitimacy, stability and funding, supervision and oversight, training and professional development, role clarity and boundaries, and access to a network of community resources. Participants reported that organizations can constrain peer work, however, and described a number of ways in which this can be avoided. A focus on peer success means that organizations structure peer work and operate in such a way that peers can make their unique contributions. In other words, organizations should be intentionally, proactively supportive (rather than merely tolerant) of peer work. Examples include trusting and respecting peers, allowing flexibility in peer work, defining roles so peers can add maximum value, investing in peer wellness and mental health, offering regular and proactive supervision, and establishing a feedback loop so peers and others can learn from each other and improve over time. One participant reported his organization conducted midyear training when,

we revisit those peer skills but then we also spend a lot of time kind of going back and checking in with members and having some guided conversations about what your biggest struggles…. What are things we can do? How can we help you, support you?

Participants repeatedly emphasized that organizations have a critical role to play in the success of veteran peers.

### Engage veterans in the community

Participants reported making efforts in the broader community to establish a presence, conduct outreach, and mobilize community members to support veterans were essential to peer work. Working in and with the community is a key component of peer work in this model as peers work with systems beyond the micro‐level. Three subcomponents were identified: show up, respect the individual, and take the long view. Participants emphasized peers must be visible in general and in veteran‐specific communities, integrating into existing structures and programs that veterans will interact with, such as student groups or veteran service organizations. For example, one participant described their organization's presence: “you can't miss it. Big old red, white, and blue [vehicle] with stuff on it and you see it when we park it in these little villages.” Peers meet veterans where they are (psychologically and geographically), help veterans feel safe, and take cues regarding timing and pace from veterans. Each veteran is an individual and peers take care to assess and understand veterans’ unique identities, circumstances, and needs. Peers approach relationship building with the veteran and community with a long‐term view, rejecting a transactional approach or narrow mental health problem focus.

### Build trusting relationships

Participants described what trusting relationships looked like and how to build them. Three subcomponents were identified: put in the work/time/effort, find shared experiences, and work together with integrity. Persistence is a hallmark of peer work as peers maintain respectful contact while showing veterans they are important. One participant described peers’ persistence as conveying the message,

I’m here, I am here with you, I’m not going to leave you while you're feeling this way. We're going to find a way to get you the help that you need. And I’m not going to quit on you.

Peers attempt to increase credibility through a caring, anti‐stigma, and proactive approach. Shared experiences are a cornerstone of peer–veteran relationships. Military service, cultural understanding, and recovery are all examples of shared experiences. Participants reported mutual trust is essential to peer relationships. Confidentiality should be clearly explained and maintained by peers. Participants agreed that confidentiality ended with the risk of danger to self and others, not unlike the limits of licensed mental health professionals. One participant reported he would not break confidentiality to report a non‐violent crime or substance use, rather he would attempt to help the veteran not to engage in this behavior. Peers show respect through a person‐centered approach, consistent follow‐through, and integrity.

### Promote connection

Participants described connecting veterans to the community. Two subcomponents were identified: create opportunities to connect and meaningful connection. Peers offer veterans opportunities to comfortably engage with other veterans and the broader community. Examples include service projects, coffee socials, and family gatherings. It is important for peers to be flexible and find approaches that appeal to veterans fit the community context. One participant commented, “you have to know what they want, know what they enjoy and then offer that to them in a completely barrier‐free way.” Peers can get creative and offer opportunities that fit the resources available. These opportunities add meaning to veterans’ lives and draw out isolated or struggling veterans before things reach a crisis level. Veterans’ families are also included as they benefit from strengthened connections to their communities, especially during times of transition or other challenges.

### Link with resources

Tangible connection to different resources is one way—alone or in parallel with promoting connection—peers were described as working to prevent suicide and effect positive change for veterans. Participants discussed navigating systems as a key element of resource connection. For example, because of their lived experience peers appear well‐suited to getting new or additional VA benefits, accompanying veterans to appointments, following up and ensuring continuity of care, and navigating lines of authority or power structures. Other important aspects of this role include knowing the community context and resources available and making timely referrals to trusted resources. Peers must be able to identify which needs can be better addressed by other resources and communicate that to veterans. As one participant exemplified, “I need to send you to an attorney because that sounds like a legal matter. I need to send you to a mental health professional because that sounds like you might need some medication.” Participants mentioned that effective resource linking can increase access to resources as well as help veterans fully use (i.e., get the most out of) existing resources.

### Veteran wellness

A wide range of positive outcomes for veterans were viewed as possible to achieve from peer work. Participants described the goals of peer work in holistic terms as a sense of overall wellness and quality of life. As one participant stated, it may involve recognizing suicide as a process that peers can interrupt,

to me suicide, in a way, is a broader, I look at it in a more broader view…to me it's about the wellness of the individual we're working with and helping that person travel through to get to that point where now he's got that journey of wellness going on, which then allows him to actually live a very productive life.

Several examples of practical and behavioral indicators of wellness were given, including taking care of self and family, having good relationships, trusting others and being trustworthy/reliable, aligning words with actions, and being able to move forward in life without crisis. Six subcomponents were identified within this component: recovery, restored community connection, renewed meaning/purpose, improved mental health/wellness, achieved personal goals, and suicide‐specific outcomes. Participants described *recovery* as a positive outcome and reported that peers may help facilitate this by sharing their journeys, role modeling, and helping to remove stigma around asking for and receiving help. Peers can also help *restore community connection* by building community, working to reengage veterans and increase community participation. Participants emphasized that *renewed meaning*/*purpose* is critical to veteran wellness and suicide prevention. One participant recognized,

veterans, for the most part, they want to serve, they want to do this type of work, they want to be involved in it. They find great value in not just being seen as the recipients of service but actually providing services.

Restoring hope was viewed as a task for peers and described as a key ingredient to recovery. Peers can create opportunities for veterans to contribute based on their strengths and to serve their communities and establish a new service mission after the military.

In addition to standard mental health outcomes (e.g., decreased depression/anxiety), outcomes reported under *improved mental health*/*wellness* included veterans feeling safe, increasing trust in others and in resources, developing coping skills, and taking better care of themselves. Helping veterans *achieve personal goals* appeared to be a way for peers to engage veterans as well as to build trusting relationships. Participants described peers as well‐suited to helping veterans set and accomplish a number of personal goals (e.g., graduating from college, gaining employment, upgrading discharges). Finally, several *suicide*‐*specific outcomes* were mentioned including reduced suicides, increased awareness of mental health and suicide risks, identification of and intervention with veterans in crisis, and development of skills and tools (e.g., a wellness recovery action plan).

### Peer and community impacts

Participants reported the impact of peer work on peers and communities. Peers themselves experienced a number of benefits from serving other veterans. Four subcomponents were identified: develop highly effective peers, enrich experience for peers, family impacts, and organization and community impacts. With experience, peers gain skill and knowledge about how to perform well in the role. Peers experience improved resilience, better self‐care, and reduced stigma about mental health problems, and suicidal ideation help peers become a useful resource to veterans and broader communities. Peer lives can be enriched by their experiences. For example, peers may experience job or role satisfaction, an increased sense of service, or improved self‐awareness of their own journey. Peer work may also be a stepping stone to further employment or educational opportunities. One participant reported, “a lot of [peers] have moved on to continue to work in higher ed or go onto veterans’ services. They continue to kind of carry that mission forward.” Veteran families can benefit from peer work in addition, either as a result of improvements in veteran wellness or more directly, such as improved communication skills within the family system. Organizations that support peers can benefit as well, learning to better structure and support peer work and leverage networks enhanced by peers. Finally, the communities where peers work gain many benefits. For example, peers can reduce stigma about mental illness and improve awareness of veteran and military culture, as well as build opportunities for veterans to connect and serve in their communities.

### Community context

Participants emphasized that the broader environment in which peers and their organizations operate profoundly shapes their ability to work effectively and sustainably. Support from the VA appeared to be critical for successful peer work and took many forms, including provider willingness to work with community‐based peers, general capacity/willingness to engage communities and share power, and financial support for peer work. Other community aspects described as important were resources available and the extent to which they are interconnected or fragmented, the embeddedness of peers’ organizations in the broader community network, and community settings where veterans and their families can feel safe and interact.

### Military culture

When asked about military culture, participants described cultural competence and sensitivity as important in all aspects of peer work, but stressed that individuals without military experience were able to serve effectively as “peers” too depending on their experiences and personal qualities (e.g., empathy, willingness to learn and listen). Cultural components of military service, such as shared vocabulary, traditions, values, and unique experiences, were agreed to be foundational to peer relationships with veterans. Shared culture provided a starting point for deep connections, as one participant stated, “with a veteran I can go sit down and I, I can talk about things with a veteran that I just wouldn't talk about with some other people.” Participants reported peers must understand nuances of the breadth of military experience and that these experiences had different meaning to each veteran. Values, such as service, teamwork, and perseverance, instilled during military service, were reported as shared across all who served. One participant conveyed the value of peers finding common ground and being respectfully inclusive as, “it's not a matter of finding the smallest box that you share with the individual but with finding the shared box.” While shared experience of serving in the military was identified as important by all participants, some also felt that sharing other aspects of service like branch of service, combat experience, service era, or particular jobs enriched the peer relationship.

## DISCUSSION

The model we developed is the first we know of to focus on veteran peers working in communities to prevent veteran suicide. Consistent with calls to study the unique contributions of peers in unique roles (Davidson et al., 2012), we learned from community programs that defined “veteran peers” broadly and “suicide prevention” holistically. Foundational elements in the peer mental health literature (e.g., building trusting relationships, connecting to resources) are embedded within military and community contexts that highlight the unique aspects of veteran peer suicide prevention. A notable difference in the peers we studied is that they were defined as “peers” on the basis of shared military experience and not solely because of shared mental illness or substance abuse problems. This allowed them to relate through a shared cultural identity and allowed them to harness military culture and build on existing strengths to promote wellness and, ultimately, prevent suicide. For example, veteran peers infuse military culture through their work and aim to prevent suicide by restoring hope and connection among veterans. In this way, suicide prevention is one of many positive outcomes peers can help achieve by working to improve veteran wellness overall. An important caveat here is that not all veterans positively identify with military culture. For example, survivors of military sexual trauma or veterans who were less than honorably discharged may benefit from working with peers who are functioning well despite sharing some of these negative lived experiences. This underscores that veteran and non‐veteran peer support services need to assess—rather than assume—the importance of matching peers on salient dimensions of military culture.

Veterans may feel isolated or disconnected after service, which puts them at risk for a host of negative outcomes. For example, unmet need for social connectedness is predictive of suicidal ideation and attempts, a central construct of Joiner's Interpersonal Theory of Suicide (Van Orden et al., 2010). Researchers have found support for this theory in veteran and military subpopulations (Rogers et al., 2017; Silva et al., 2017; Wolfe‐Clark & Bryan, 2017). Its two dimensions, loneliness and absence of reciprocal–caring relationships (Van Orden et al., 2010), were frequently mentioned by participants as concerns for veterans benefiting from their peer work. We heard participants describe functions of community peer work, specifically in the “build trusting relationships” and “promote connection” components, which address these dimensions. Through meaningful peer relationships, veterans may be able to ameliorate these problems and exit the causal pathway to suicide. Participants also discussed the development of “renewed meaning and purpose” as a subcomponent of the “veteran wellness component.” We see this outcome as possibly reducing another central construct of Joiner's theory, “perceived burdensomeness” (Van Orden et al., 2010), offering another mechanism for suicide prevention.

Participants described different organizational and community characteristics critical to the success of veteran peer suicide prevention, including proactive supervision, clear job and role descriptions, and integration within a network of community resources. These findings likely apply to non‐veteran peer support services as well, given that they point to broader contextual conditions impacting the ability of peers to add their unique value. Literature on peer support acknowledges that institutional cultural change is needed to integrate peers within healthcare organizations (Chinman et al., 2010; Davidson et al., 2012; Hebert et al., 2008) but pays relatively little attention to these broader contextual factors that appear to shape the reach, impact, and sustainability of community‐based veteran peer suicide prevention efforts. More research is needed to understand the organizational and community conditions under which veteran—and non‐veteran—peer suicide prevention initiatives are most effective.

Our conversations with participants led us to believe that there are many conditions under which peers can be effective and that peer ability to respond to local needs and circumstances is essential. Participants referred to this as a “yellow lines” approach in which peers are given guidelines and boundaries, but allowed to operate flexibly within them to address veteran needs and priorities as they arise. This has implications for how peer work is structured, managed, and supported within organizations, and suggests that raising awareness among different stakeholders of what peers do and how they can best add unique value is important. For example, peers may need to work flexible hours to be most effective. If others (e.g., peer supervisors, community providers) do not understand or support such flexibility, peers may be limited in their ability to help other veterans. Again, these findings likely apply to other non‐veteran peer support services in which flexibility and creativity are needed to identify, connect with, and support other populations that are “hard to reach” because of structural or cultural barriers.

Extensive input from veteran peers and peer program managers strengthened this study, though there were limitations worth noting. We conducted seven interviews, which gave us in‐depth understanding of six community‐based veteran peer suicide prevention programs. It is unclear, however, the extent to which findings would apply to other similar efforts. In addition, because we sampled from contacts of the Together With Veterans program, which has a rural focus, it is not clear how findings would hold across different geographic locations. When asked directly about this, participants felt that the model was comprehensive though specific factors (e.g., surrounding community resources) might impact peer effectiveness more directly depending on location. The “yellow lines” approach of this model leaves individual organizations with significant flexibility for developing their own programs. For example, organizations would decide the type of training, supervision, and certification needed by their peers as well as how to address issues of liability.

This model identified a range of outcomes of community‐based veteran peer suicide prevention that is broader than typically conceived. Future research would benefit from measuring wellness‐oriented or strengths‐based outcomes (e.g., hope, community participation, sense of belonging/connection) and outcomes at multiple levels (e.g., impacts on peers and families) in addition to more traditional, deficit‐based outcomes (e.g., depression, suicidal ideation). Additionally, the order and sequencing of these outcomes are not yet clear. In‐depth case studies or longitudinal studies may be useful for identifying which are short‐, medium‐, and long‐term outcomes. Finally, the “yellow lines” approach has implications for program implementation and research. Since flexibility and responsiveness are critical to peer veteran suicide prevention, understanding how and whether such programs adhere to guiding principles and critical processes may be more important than measuring strict “fidelity” to a prescribed set of steps (Hawe et al., 2004). Principles‐focused evaluation is one way in which this can be accomplished (Patton, 2017).

## DISCLAIMERS

The contents do not represent the views of the U.S. Department of Veterans Affairs or the United States Government.
